# Racial and socioeconomic disparities in survival among patients with metastatic non–small cell lung cancer

**DOI:** 10.1093/jnci/djae118

**Published:** 2024-06-03

**Authors:** Dipesh Uprety, Randell Seaton, Tarik Hadid, Hirva Mamdani, Ammar Sukari, Julie J Ruterbusch, Ann G Schwartz

**Affiliations:** Department of Medical Oncology, Barbara Ann Karmanos Cancer Institute, Wayne State University, Detroit, MI, USA; Population Studies and Disparities Research Program, Barbara Ann Karmanos Cancer Institute, Wayne State University, Detroit, MI, USA; Department of Medical Oncology, Barbara Ann Karmanos Cancer Institute, Wayne State University, Detroit, MI, USA; Department of Medical Oncology, Barbara Ann Karmanos Cancer Institute, Wayne State University, Detroit, MI, USA; Department of Medical Oncology, Barbara Ann Karmanos Cancer Institute, Wayne State University, Detroit, MI, USA; Population Studies and Disparities Research Program, Barbara Ann Karmanos Cancer Institute, Wayne State University, Detroit, MI, USA; Population Studies and Disparities Research Program, Barbara Ann Karmanos Cancer Institute, Wayne State University, Detroit, MI, USA

## Abstract

**Background:**

Immune checkpoint inhibitors have profoundly impacted survival among patients with metastatic non–small cell lung cancer. However, population-based studies evaluating this impact on survival by race and socioeconomic factors are lacking.

**Methods:**

We used the Surveillance, Epidemiology, and End Results Program–Medicare database to identify patients with metastatic non–small cell lung cancer diagnosed between 2015 and 2019. The primary study outcomes were the receipt of an immune checkpoint inhibitor and overall survival. χ^2^ tests and logistic regression were used to identify demographic factors associated with receipt of immune checkpoint inhibitors. The Kaplan–Meier method was used to calculate 2-year overall survival rates, and log-rank tests were used to compare survival by race and ethnicity.

**Results:**

Of 17 134 patients, approximately 39% received an immune checkpoint inhibitor. Those diagnosed with cancer recently (in 2019); who are relatively younger (aged younger than 85 years); non-Hispanic White, non-Hispanic Asian, or Hispanic; living in high socioeconomic status or metropolitan areas; not Medicaid eligible; and with adenocarcinoma histology were more likely to receive immune checkpoint inhibitors. The 2-year overall survival rate from diagnosis was 21% for the overall population. The 2-year overall survival rate from immune checkpoint inhibitor initiation was 30%, among those who received at least 1 cycle and 11% among those who did not receive immune checkpoint inhibitors. The 2-year overall survival rates were higher among non-Hispanic White (22%) and non-Hispanic Asian (23%) patients compared with non-Hispanic Black (15%) and Hispanic (17%) patients. There was no statistically significant racial differences in survival for those who received immune checkpoint inhibitors.

**Conclusion:**

Immune checkpoint inhibitor utilization rates and the resulting outcomes were inferior for certain vulnerable groups, mandating the need for strategies to improve access to care.

Lung cancer is the most common cause of cancer-related deaths worldwide (1). Although platinum-based chemotherapy has been the mainstay of treatment for many decades, considerable advancements have been achieved in treating non–small cell lung cancer (NSCLC) in recent years. These include targeting the mutant protein with small molecule tyrosine kinase inhibitors and using immune checkpoint inhibitors. A population-based study utilizing data from the Surveillance, Epidemiology, and End Results (SEER) database showed a sharp decline in population-level mortality from NSCLC in the United States from 2013 to 2016 (2). This decline in mortality is partly related to the increase in the use of targeted therapies in oncogene-addicted NSCLC. Although targeting the mutant protein is the treatment of choice for oncogene-addicted NSCLC, most tumors, particularly in older patients, do not carry a known driver mutation. For these patients, immune checkpoint inhibitor, with or without platinum-based chemotherapy, is now the treatment of choice. CheckMate-017 and CheckMate-057 are phase 3 trials that randomly assigned patients with squamous and nonsquamous NSCLC, respectively, to receive either nivolumab or docetaxel and showed significant survival benefits from immune checkpoint inhibitors (3-5). Based on the results of these trials, nivolumab was the first immune checkpoint inhibitor approved by the US Food and Drug Administration (FDA) on March 4, 2015, for the treatment of stage IV NSCLC patients in the second-line setting (6). Additionally, pembrolizumab (for patients with programmed cell death 1 ligand positivity ≥1%) and atezolizumab were FDA approved in 2016. Subsequently, immune checkpoint inhibitor monotherapy or in combination with chemotherapy was FDA approved in first-line settings for treatment of stage IV NSCLC without a driver mutation. Although immune checkpoint inhibitors have profoundly improved survival in patients with stage IV NSCLC without a driver mutation, population-based studies to assess the utilization of immune checkpoint inhibitors are lacking. Additionally, studies evaluating the impact of immune checkpoint inhibitors on survival based on race are lacking. Historically, there have been significant racial disparities in receiving recommended treatment for patients with NSCLC (7). In this study, we sought to evaluate racial differences in immune checkpoint inhibitor treatment and corresponding survival of patients with metastatic NSCLC in the immunotherapy era utilizing the SEER-Medicare database.

## Methods

### Data source

We used data from the SEER-Medicare database, a high-quality data source reflecting the linkage of 2 population-based sources, which provides detailed information about elderly patients with cancer in the United States. The SEER Program, sponsored by the National Cancer Institute, is considered the gold standard for cancer registries worldwide and is the only source for population-based cancer data in the United States that includes patient survival information. SEER has collected cancer incidence and survival data since 1973 and now is composed of 22 population-based cancer registries covering approximately 48% of the US population (8). Medicare is the primary health insurer for 97% of the US population aged 65 years and older. Through linking SEER registry data to Medicare enrollment and claims information for patients diagnosed in the SEER regions, the SEER-Medicare database provides detailed information on treatment and outcomes of elderly patients diagnosed with cancer.

### Study population

**Figure 1.**
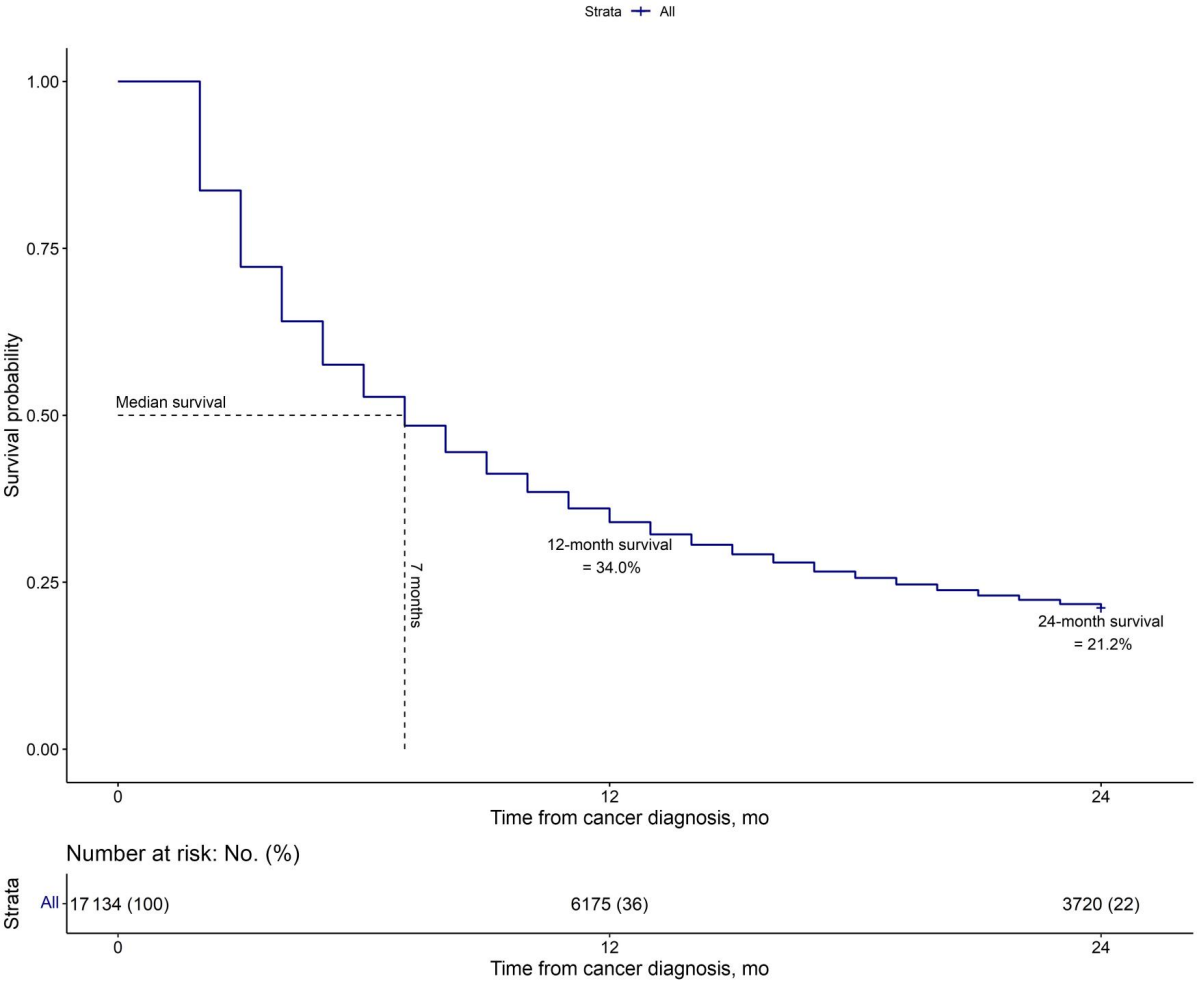
Two-year overall survival of the entire population.

We included SEER-Medicare patients diagnosed with primary invasive NSCLC (site code C340-C349, histology codes by group—squamous: 8051-52, 8070-76, 8078, 8083-84, 8090, 8094, 8120, and 8123; adenocarcinoma: 8015, 8050, 8140-41, 8143-45, 8147, 8190, 8201, 8211, 8250-55, 8260, 8290, 8310, 8320, 8323, 8333, 8401, 8440, 8470-71, 8480-01, 8490, 8503, 8507, 8550, 8570-72, 8574, and 8576; large cell: 8012-14, 8021, 8034, and 8082; NSCLC, not otherwise specified: 8046; other specified carcinomas: 8003-04, 8022, 8030-33, 8035, 8200, 8240-41, 8243-46, 8249, 8430, 8525, 8560, 8562, and 8575; *International Classification of Diseases for Oncology 3rd Edition*) diagnosed with metastatic disease (combined SEER summary stage = distant) (9,10). Because nivolumab, an immune checkpoint inhibitor, was approved by the FDA in March 2015, we only included patients diagnosed between January 1, 2015, and December 31, 2019 (the most recent year available from the SEER-Medicare program at the time). All patients were aged 65 years and older at the time of cancer diagnosis and enrolled in Medicare parts A, B, and D from the time of their cancer diagnosis until death or through December 2020, the end of study observation. Those enrolled in a Medicare health maintenance organization were excluded to avoid the potential for missing claims data. In addition, patients who died within 1 month of their cancer diagnosis were excluded. Because tyrosine kinase inhibitors are recommended for patients with metastatic NSCLC with sensitizing driver mutation, and because immune checkpoint inhibitors provide modest to no clinical benefit for these patients, those who filled a prescription for a tyrosine kinase inhibitor after their lung cancer diagnosis were also excluded from the study (see Figure 1). All patients were followed after cancer diagnosis until death or the end of study observation. The study was reviewed and approved by the Wayne State University institutional review board as nonhuman participant research.

### Study measures

The main study outcome was the receipt of an immune checkpoint inhibitor, which was defined as having at least 1 Medicare claim with a Healthcare Common Procedure Coding System code for the following drugs: pembrolizumab (J9271, C9027), nivolumab (J9299, C9453), atezolizumab (J9022, C9483), durvalumab (J9173, C9492), ipilimumab (J9228, C9284), or cemiplimab (J9119). Each Medicare claim corresponds to 1 cycle of treatment. In addition, Medicare claims were used to identify patients who received chemotherapy and radiation therapy using codes recommended by the SEER-Medicare program (11). Vital status and survival time were obtained from the Medicare enrollment files, and survival time was calculated from diagnosis to death or end of study observation. For the group who received an immune checkpoint inhibitor, survival time was also calculated from the date of receiving their first immune checkpoint inhibitor (the day of the immune checkpoint inhibitor claim) to death or end of study observation. Histology codes were categorized into 3 groups: squamous, adenocarcinoma, and “others,” which include large cell, NSCLC, not otherwise specified, and other specified carcinoma using codes previously referenced. Medicare-Medicaid dual eligibility was operationalized among Medicare beneficiaries as having full and/or partial Medicaid (Medicare Savings Programs and/or full Medicaid benefits) within the same month and year of cancer diagnosis. Other variables included in this analysis were taken from SEER files and include year of cancer diagnosis, sex, age at cancer diagnosis, race and ethnicity, *Yost* index for socioeconomic status (SES), and urban and rural indicator.

### Statistical analysis

Statistical analyses were performed using the Statistical Analysis Systems software package (V.9.4; Cary, NC, USA) and R Studio version 4.3.2 to illustrate Kaplan–Meier curves. Descriptive statistics were used to assess characteristics of the overall population along with the number of immune checkpoint inhibitor claims and time to first immune checkpoint inhibitor claim (or time-to-treatment initiation) among participants who have received an immune checkpoint inhibitor. A general χ^2^ test was used to identify demographic and cancer-related variables significantly associated with receiving immune checkpoint inhibitors. Additionally, logistic regression was used to calculate odds ratios and 95% confidence intervals adjusted for variables statistically significant (α = 0.05) in univariate analysis. The Kaplan–Meier method was used to calculate 2-year survival estimates and generate overall survival curves for all patients and stratified by race. Differences in overall survival were evaluated using log-rank tests.

## Results

A total of 17 134 patients who met the inclusion criteria were identified in the SEER-Medicare database between 2015 and 2019 (CONSORT diagram in Supplementary Figure 1, available online). The demographic characteristics of the patients are summarized in Table 1. The median age of patients was 74 years (range = 65-100 years). There was no gender predilection; most patients were White (81.3%), lived in a metropolitan area (82.7%), and had adenocarcinoma histology (60.7%). Approximately 39% (n = 6634) of the patients received an immune checkpoint inhibitor within the study period, with the utilization rate increasing from 21.9% in 2015 to 55.4% in 2019 (*P* < .001; Table 2). Among the 6634 patients who received immune checkpoint inhibitors, there appeared to be no difference in the mean number of immune checkpoint inhibitor cycles by race. However, the mean time to immune checkpoint inhibitor initiation appeared to differ among different racial groups (Supplementary Table 1, available online).

**Table 1.** Characteristics of metastatic non–small cell lung cancer patients diagnosed from 2015 to 2019 identified from Surveillance, Epidemiology, and End Results–Medicare files^a^

Characteristics	No. (%)
Total, all patients	17 134 (100)
Year of cancer diagnosis	
2015	3763 (22.0)
2016	3416 (19.9)
2017	3470 (20.3)
2018	3229 (18.9)
2019	3256 (19.0)
Sex	
Male	8565 (50.0)
Female	8569 (50.0)
Age group, y	
65-69	4331 (25.3)
70-74	4544 (26.5)
75-79	3844 (22.4)
80-84	2618 (15.3)
85 and older	1797 (10.5)
Mean (SD)	75.0 (6.9)
Median (range)	74 (65-100)
Race and ethnicity	
Hispanic, all races	934 (5.5)
Non-Hispanic American Indian and Alaska Native	61 (0.4)
Non-Hispanic Asian or Pacific Islander	788 (4.6)
Non-Hispanic Black	1403 (8.2)
Non-Hispanic White	13 922 (81.3)
Non-Hispanic unknown race	26 (0.2)
*Yost* index state quintiles (area level SES deprivation)	
Group 1, lowest SES	2905 (17.0)
Group 2, low-middle SES	3462 (20.2)
Group 3, middle SES	3627 (21.2)
Group 4, high-middle SES	3419 (20.0)
Group 5, highest SES	3285 (19.2)
Unknown, blank	436 (2.5)
Medicare-Medicaid dual eligibility	
Yes	4083 (23.8)
No	13 051 (76.2)
Urban rural indicator	
Metropolitan	14 166 (82.7)
Nonmetropolitan	2968 (17.3)
Non–small cell histology	
Squamous	4491 (26.2)
Adenocarcinoma	10 400 (60.7)
Other	2243 (13.1)
Treatment	
Immune checkpoint inhibitor, with or without chemotherapy and/or radiation	6634 (38.7)
Chemotherapy and radiation	2731 (15.9)
Chemotherapy only	1540 (9.0)
Radiation only	2467 (14.4)
None of above	3762 (22.0)

a SES = socioeconomic status.

**Table 2.** Unadjusted and adjusted odds ratios of demographic data for patients who received immune checkpoint inhibitor treatment

Immune checkpoint inhibitor claim						
	Row %	No., yes	No., no	*P*	OR (95% CI)	AOR^a^ (95% CI)
Total, all patients	38.7	6634	10 500			
Year of cancer diagnosis				<.001		
2015	21.9	825	2938		Referent	Referent
2016	29.9	1023	2393		1.52 (1.37 to 1.69)	1.52 (1.36 to 1.69)
2017	39.0	1353	2117		2.28 (2.05 to 2.52)	2.34 (2.11 to 2.61)
2018	50.4	1628	1601		3.62 (3.27 to 4.02)	3.76 (3.37 to 4.18)
2019	55.4	1805	1451		4.43 (3.99 to 4.91)	4.64 (4.17 to 5.17)
Sex				.104		
Male	39.3	3368	5197		1.05 (0.99 to 1.12)	1.06 (0.99 to 1.133)
Female	38.1	3266	5303		Referent	Referent
Age group, y				<.001		
65-69	43.3	1876	2455		2.47 (2.18 to 2.79)	3.09 (2.71 to 3.53)
70-74	43.6	1983	2561		2.50 (2.21 to 2.83)	2.89 (2.53 to 3.29)
75-79	39.4	1516	2328		2.10 (1.85 to 2.39)	2.33 (2.03 to 2.66)
80-84	31.9	834	1784		1.51 (1.32 to 1.73)	1.57 (1.36 to 1.81)
85 and older	23.7	425	1372		Referent	Referent
Mean (SD)		73.9 (6.2)	75.8 (7.2)			
Race and ethnicity				<.001		
Hispanic, all races	34.0	318	616		1.29 (1.08 to 1.55)	1.45 (1.20 to 1.76)
Non-Hispanic American Indian and Alaska Native	24.6	15	46		0.82 (0.45 to 1.48)	0.69 (0.37 to 1.32)
Non-Hispanic Asian or Pacific Islander	37.3	294	494		1.49 (1.24 to 1.80)	1.55 (1.26 to 1.90)
Non-Hispanic Black	28.5	400	1003		Referent	Referent
Non-Hispanic White	40.2	5590	8332		1.68 (1.49 to 1.90)	1.36 (1.19 to 1.56)
*Yost* index state quintiles, area level SES deprivation				<.001		
Group 1, lowest SES	30.8	895	2010		Referent	Referent
Group 2, low-middle SES	36.2	1253	2209		1.27 (1.15 to 1.42)	1.14 (1.02 to 1.27)
Group 3, middle SES	41.3	1400	2227		1.41 (1.27 to 1.57)	1.21 (1.08 to 1.35)
Group 4, high-middle SES	44.0	1427	1992		1.61 (1.45 to 1.79)	1.30 (1.16 to 1.47)
Group 5, highest SES	44.8	1472	1813		1.82 (1.64 to 2.02)	1.49 (1.32 to 1.68)
Medicare-Medicaid dual eligibility				<.001		
Yes	28.5	1163	2920		Referent	Referent
No	41.9	5471	7580		1.81 (1.68 to 1.96)	1.76 (1.62 to 1.93)
Urban rural indicator				<.001		
Metropolitan	39.3	5572	8594		1.16 (1.07 to 1.26)	1.13 (1.02 to 1.24)
Nonmetropolitan	35.8	1062	1906		Referent	Referent
Non–small cell histology				<.001		
Squamous	35.9	1613	2878		Referent	Referent
Adenocarcinoma	41.3	4296	6104		1.26 (1.17 to 1.35)	1.19 (1.10 to 1.28)
Other	32.3	725	1518		0.85 (0.77 to 0.95)	0.77 (0.69 to 0.87)

a Odds ratios are adjusted for all variable statistically significant in univariate analysis (*P* < .05). Row % = Yes/(Yes+No). CI = confidence interval; AOR = adjusted odds ratio; OR = odds ratio; SES = socioeconomic status.

**Figure 2.**
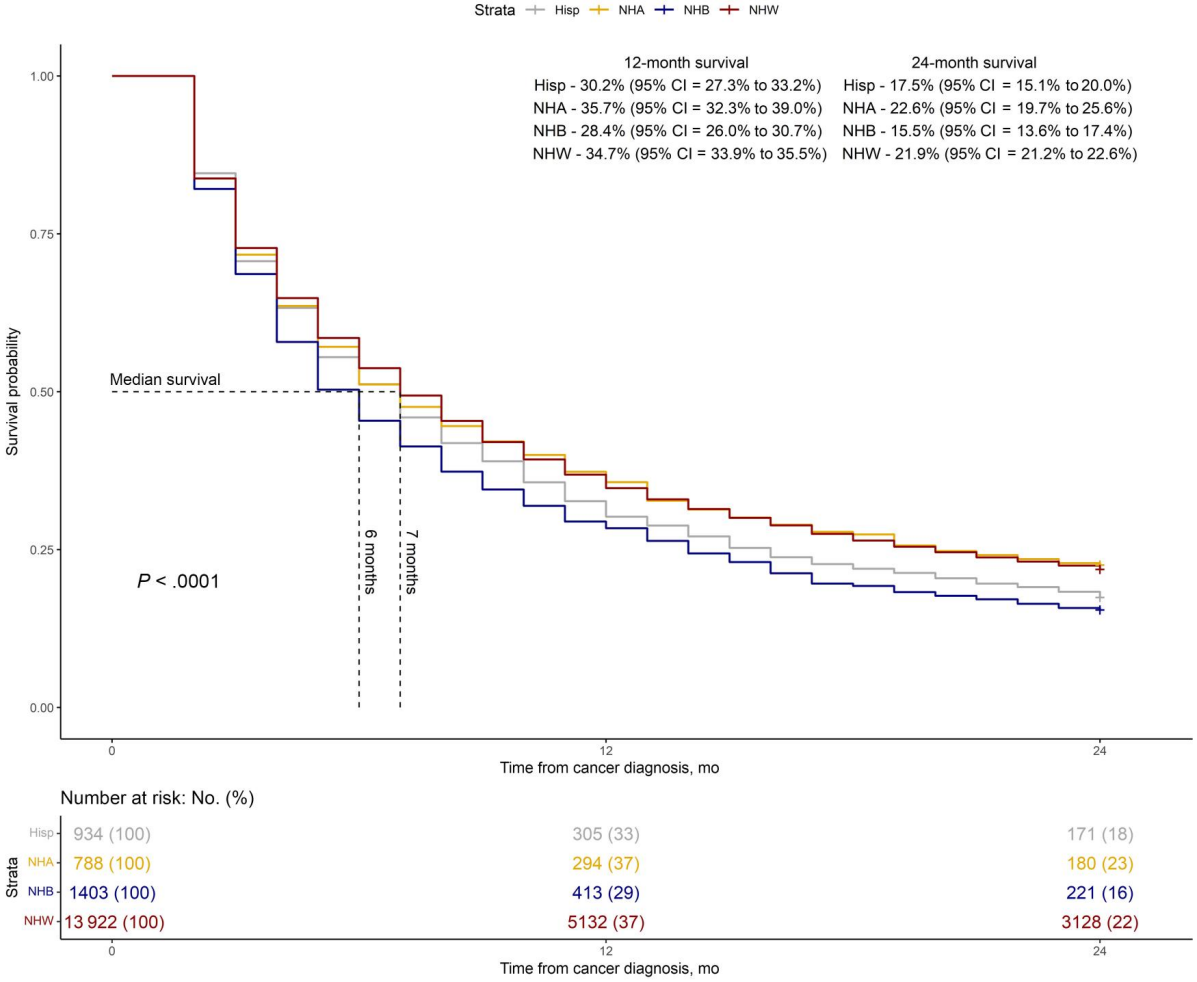
Two-year overall survival of the entire population by racial-ethnic group. CI = confidence interval; Hisp = Hispanic; NHA = non-Hispanic Asian; NHB = non-Hispanic Black; NHW = non-Hispanic White.

In the overall population, the median overall survival was 7 months, and the 1-year and 2-year overall survival rates were 34% and 21%, respectively (Figure 1). The 2-year overall survival rates were higher for White (22%) and Asian (23%) patients when compared with Black (15%) and Hispanic (17%) patients (Figure 2; log rank *P* <.001).

**Figure 3.**
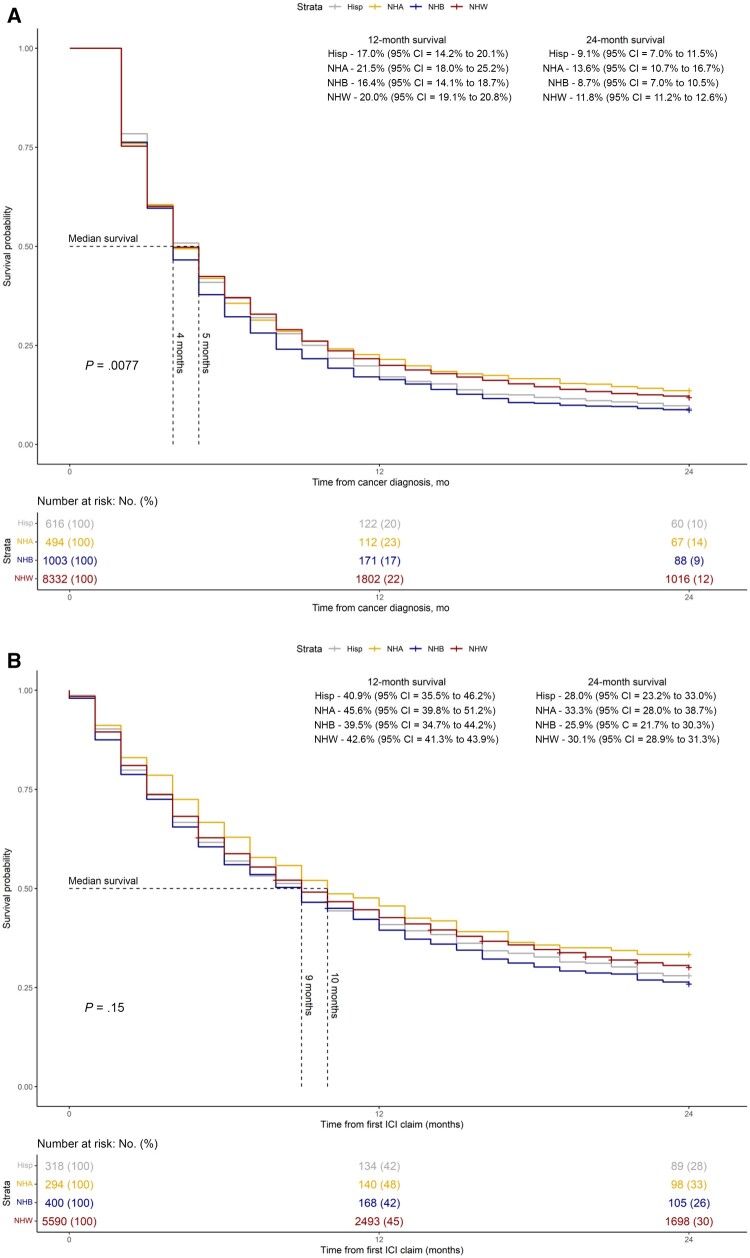
**A)** Two-year overall survival beginning from cancer diagnosis by racial-ethnic group among patients who did not receive immune checkpoint inhibitors. **B)** Two-year overall survival beginning from first immune checkpoint inhibitor claim by racial-ethnic group among patients who received at least 1 immune checkpoint inhibitor cycle. CI = confidence interval; Hisp = Hispanic; ICI = immune checkpoint inhibitor; NHA = non-Hispanic Asian; NHB = non-Hispanic Black; NHW = non-Hispanic White.

For those who did not receive immune checkpoint inhibitors, the median overall survival was 4 months, and the 1-year and 2-year overall survival rates were 19.5% and 11.4%, respectively (Figure 3, A). The 2-year overall survival rates were 12% for White and 14% for Asian patients when compared with 9% for Black and Hispanic patients.

There was a statistically significant difference in the receipt of an immune checkpoint inhibitor by race (*P* < .001) with only 28.5% of non-Hispanic Black and 24.6% of American Indian and Alaska Native patients receiving immune checkpoint inhibitors compared with 40.2% for non-Hispanic White patients. In multivariable analysis, recent cancer diagnosis year; younger patients; being a non-Hispanic White, non-Hispanic Asian, or Hispanic (compared with non-Hispanic Black) individual; those living in high SES areas; those who are not Medicaid eligible; those living in a metropolitan area; and those diagnosed with adenocarcinoma were more likely to receive immune checkpoint inhibitors (Table 2).

Among those who received at least 1 cycle of an immune checkpoint inhibitor, the median overall survival, 1-year, and 2-year overall survival rates were 9 months, 42.6%, and 29.9%, respectively. Furthermore, patients who received at least 2 cycles of an immune checkpoint inhibitor had a median overall survival, 1-year, and 2-year overall survival rates of 12 months, 48.7%, and 34.3%, respectively. Additionally, the differences in survival by race were not statistically significant for patients who received at least 1 cycle of immune checkpoint inhibitors and for those who received 2 or more immune checkpoint inhibitor cycles (Figure 3, B;Supplementary Figure 2, available online), with 2-year overall survival rates of 30% for White and 33% for Asian patients when compared with 26% for Black and 28% for Hispanic patients.

## Discussion

Immune checkpoint inhibitors have profoundly impacted survival among patients with stage IV NSCLC. Although these agents are known to improve median overall survival compared with chemotherapy, the greatest benefit of these agents is their ability to achieve a durable clinical benefit in a small subset of patients. The use of immune checkpoint inhibitors, with or without chemotherapy, improved 2-year overall survival rates to 36.0%-51.5% in the first-line setting (12-15) and to 27% in the second-line setting (3). Our study demonstrated a 2-year overall survival rate of 29.9% for those who received at least 1 cycle of immune checkpoint inhibitors, which is in concordance with the previously reported figures in major phase III clinical trials (3,12-15). Given the challenges of inferring lines of therapy from claims data, our study did not stratify the overall survival benefit of immune checkpoint inhibitors based on the lines of therapy. Additionally, for those who had received at least 1 cycle of immune checkpoint inhibitors, there appeared to be no difference in the number of immune checkpoint inhibitor cycles received among different races. Because the number of immune checkpoint inhibitor cycles received relates to the composite effect of toxicity and treatment outcome, this may suggest that the toxicity profile of immune checkpoint inhibitors may be similar among different races. Nonetheless, this observation should be interpreted with caution and requires further prospective validation. In their retrospective study, Bhandari et al. (16) also demonstrated similar immune-related adverse events between White and African American individuals.

Although the introduction of immune checkpoint inhibitors and targeted therapy has improved survival of NSCLC patients, racial and ethnic disparities in lung cancer epidemiology and outcome persist. Although the incidence of lung cancer among African American and White patients is similar (17), African American patients with lung cancer are diagnosed at a younger age, more often male, and more frequently diagnosed with distant disease compared with White patients (18). Additionally, African American patients are less likely to receive recommended treatment, including surgery and, hence, have inferior survival outcomes (17,19,20). African American patients are more likely to have a poor SES, and hence, they have a lower likelihood of having access to health-care insurance when compared with White patients. Previous studies have demonstrated that having access to health-care insurance is one of the major predictors of better cancer-related outcomes in the United States (21,22). Taking our investigation a step further, we demonstrated that the utilization of immune checkpoint inhibitors and the resulting lung cancer–related outcomes were inferior in certain vulnerable Medicare-insured populations; these groups included the extremely older population (aged 85 years and older), non-Hispanic Black individuals, non-Hispanic American Indian individuals, those with the lowest SES quintile, those who are Medicare-Medicaid dual eligible, and those living in nonmetropolitan areas. Taken together, patients with low SES and/or living in low-income neighborhoods were less likely to receive immune checkpoint inhibitors, leading to an inferior outcome. Because all the patients in our study are Medicare insured, the low utilization of immune checkpoint inhibitors and the inferior outcome are likely due to other limitations in access to care. Various studies have associated decrease access to health care within lower SES (23,24). The negative impact of these measures on survival outcomes among these vulnerable groups should alert policy makers to develop tailored population-based health interventions for lung cancer patients.

An additional interesting finding of our study is the considerable racial disparity noted in patients who did not receive immune checkpoint inhibitors. This disparity is lost when patients receive immune checkpoint inhibitors (≥1 immune checkpoint inhibitor cycle or ≥2 immune checkpoint inhibitor cycles). This suggests that the racial disparity is predominately driven by access to health care rather than biological differences, as patients, regardless of their race, who have access to immune checkpoint inhibitors have comparable overall survival without significant racial disparity, unlike those who do not have access to immune checkpoint inhibitors. A single-center retrospective study of 248 patients also demonstrated a similar benefit of immune checkpoint inhibitors among different racial and ethnic groups in patients with metastatic NSCLC (25).

This study has several strengths. To our knowledge, this is the first study to date describing racial and socioeconomic disparities in the utilization of immunotherapies and their impact on survival in patients with metastatic NSCLC at the population level. We included 17 134 Medicare patients with metastatic NSCLC, representing diverse socioeconomic backgrounds on a national scale.

This study has some limitations. First, this is an observational cohort study with the possibility of survival bias as patients with more aggressive disease may die before having the opportunity for immune checkpoint inhibitor treatment. Second, the results from this study may not accurately reflect outcomes in younger NSCLC patients, as the SEER-Medicare database only includes patients aged 65 years and older. Third, our analysis did not include specific patient-level data, such as a patient’s performance status, medical comorbidities, immune checkpoint inhibitor line of therapy, or history of autoimmune disease, which are important in predicting the clinical outcomes in NSCLC and could also influence selection of patients for immune checkpoint inhibitor therapy. Finally, the SEER-Medicare dataset doesn’t offer any insights into the treatment decision making process, as it is unknown if immune checkpoint inhibitors were recommended or offered and what other barriers may have influenced the physician and/or patient decision when considering immune checkpoint inhibitor therapy.

In summary, our retrospective study, utilizing the SEER-Medicare database, found that the utilization rate of immune checkpoint inhibitors was substantially lower among African American patients and patients with low SES and living in low-income neighborhoods, which led to inferior survival outcomes. This underscores policy makers’ need to develop targeted health interventions to increase access to care.

## Supplementary Material

djae118_Supplementary_Data

## Data Availability

This project was completed using Surveillance, Epidemiology, and End Results (SEER)–Medicare linked data. Per the required data use agreement needed to obtain this data, it cannot be made publicly available for patient confidentiality reasons. These data are available to other investigators with permission from the SEER-Medicare program for the study of specific research questions.
