# Supplementary material for: Racial and socioeconomic disparities in survival among patients with metastatic non–small cell lung cancer
Source: J Natl Cancer Inst. 2024 Jun 3;116(10):1697–704. doi: 10.1093/jnci/djae118 (PMC11461161; doi:10.1093/jnci/djae118)
Supplement: djae118_Supplementary_Data [file djae118_supplementary_data.pdf]

Supplementary Table 1: Number of ICI cycles and time to initiation of immune checkpoint inhibitor (ICI) for metastatic NSCLC patients by race/ethnicity

	N	%
**Total (patients who received ICI)**	6,634	100
**# of ICI claims overall**		
1-5	3,253	49.0
6-10	1,304	19.7
11-20	1,130	17.0
21-30	500	7.5
30+	447	6.7
	**Mean (s.d.)**	**Median (range)**
**# of ICI claims by race/ethnicity**		
**All Cases with ICI claim**	10.1 (12.0)	6 (1-111)
Non-Hispanic White	10.1 (12.0)	6 (1-111)
Non-Hispanic Black	9.8 (11.7)	5.5 (1-73)
Non-Hispanic American Indian/Alaska Native	9.0 (14.9)	2 (1-48)
Non-Hispanic Asian or Pacific Islander	10.7 (12.6)	6 (1-85)
Hispanic	9.8 (10.8)	6 (1-96)
**Time from Cancer Diagnosis to receiving first ICI claim by race/ethnicity (months)**		
**All patients who received ICI**	5.4 (6.8)	3 (0-67)
Non-Hispanic White	5.4 (7.0)	3 (0-67)
Non-Hispanic Black	5.9 (6.7)	4 (0-62)
Non-Hispanic American Indian/Alaska Native	2.5 (2.1)	2 (1-9)
Non-Hispanic Asian or Pacific Islander	4.7 (5.5)	2 (0-33)
Hispanic	5.3 (5.8)	3 (0-43)

Supplementary Figure 1: CONSORT

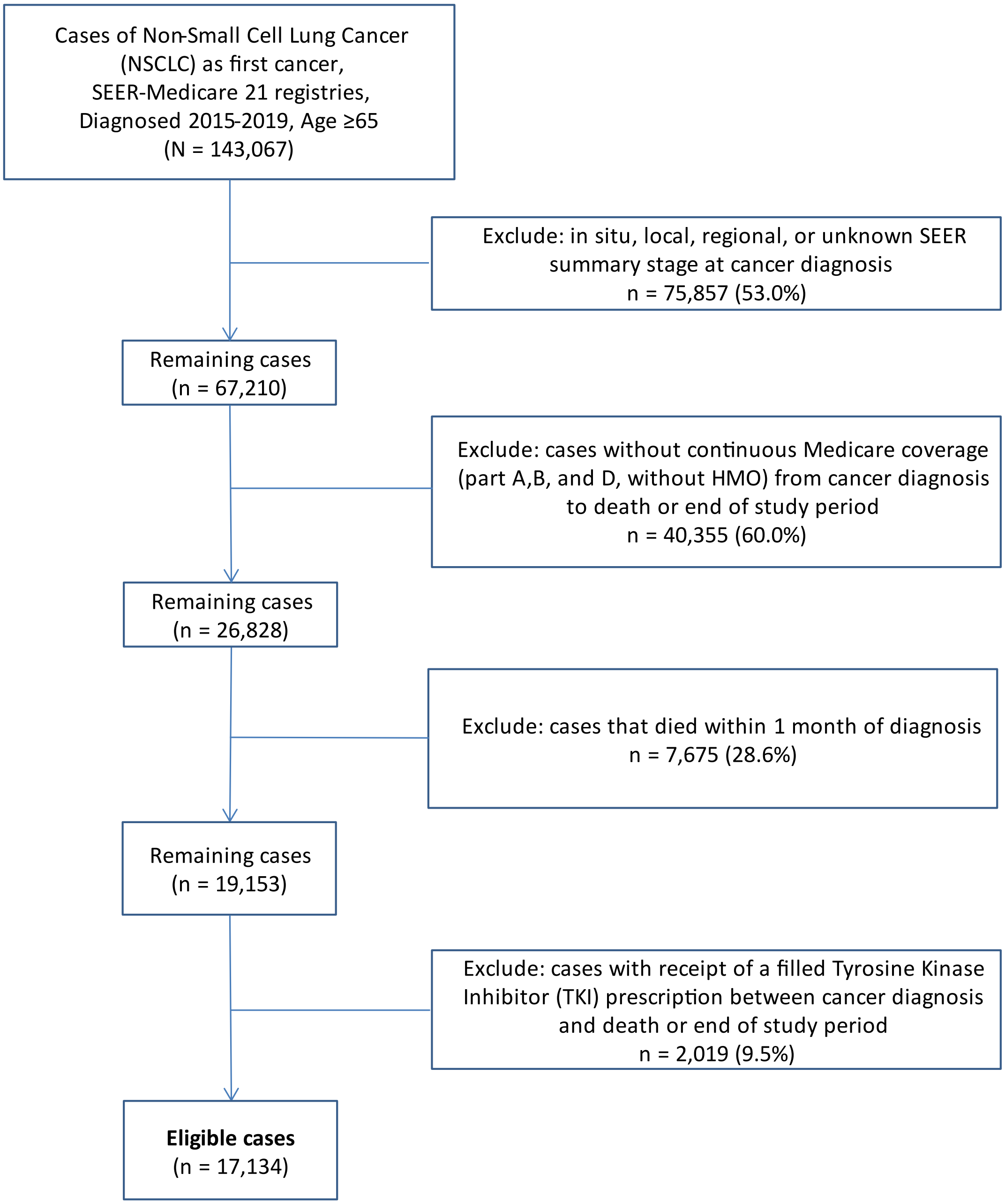

Supplementary Figure 2: 2-Year Overall Survival Beginning from First Immune Checkpoint Inhibitor (ICI Claim by Racial-Ethnic Group among Patients with 2 or more ICI claims

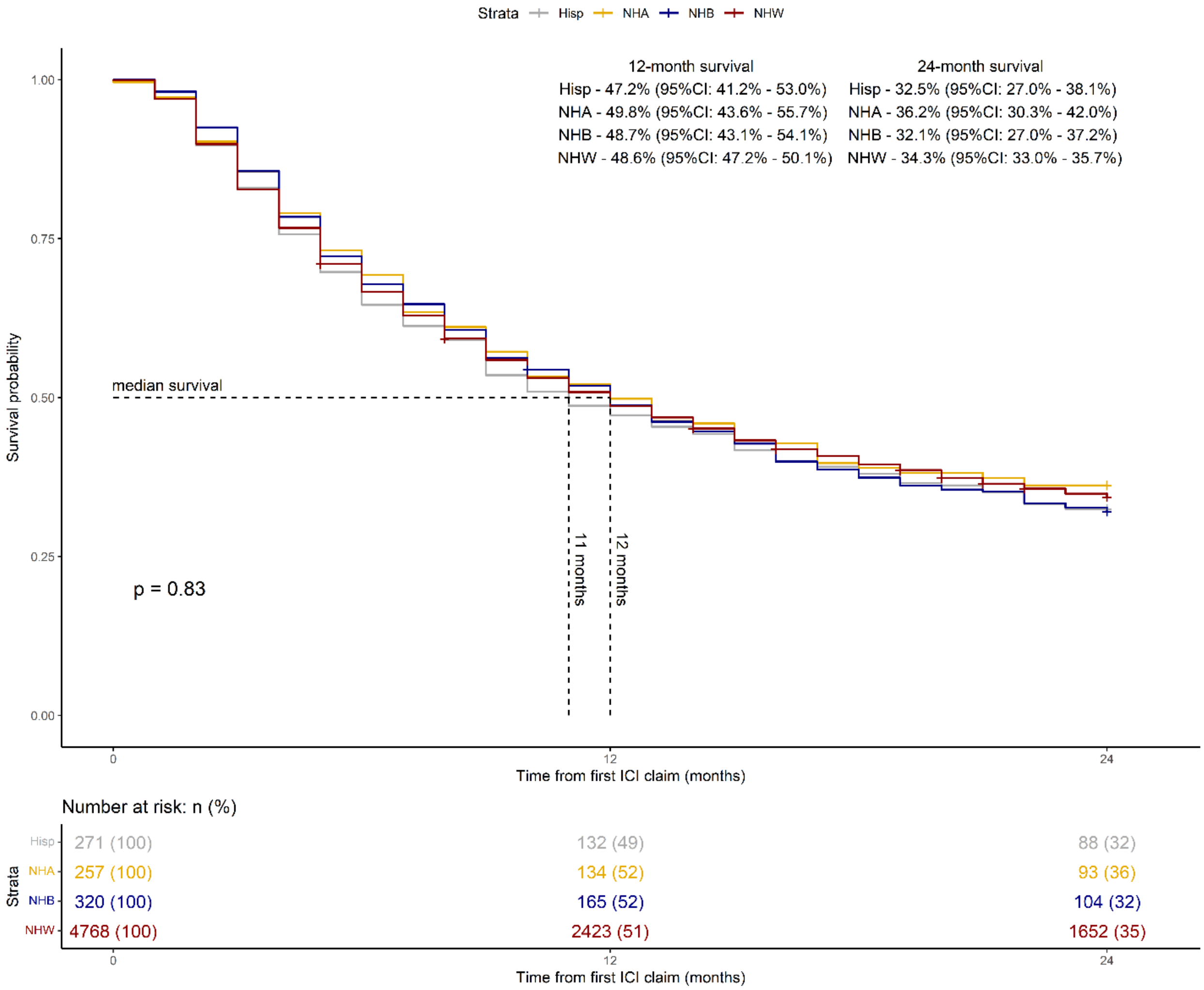

Abbreviations: Hisp = Hispanic, NHA = Non-Hispanic Asian, NHB = Non-Hispanic Black, NHW = Non-Hispanic White
